# Type 2 Diabetes and ADP Receptor Blocker Therapy

**DOI:** 10.1155/2016/6760710

**Published:** 2015-12-28

**Authors:** Matej Samoš, Marián Fedor, František Kovář, Michal Mokáň, Tomáš Bolek, Peter Galajda, Peter Kubisz, Marián Mokáň

**Affiliations:** ^1^Department of Internal Medicine I, Jessenius Faculty of Medicine in Martin, Comenius University in Bratislava, 036 59 Martin, Slovakia; ^2^National Center of Hemostasis and Thrombosis, Department of Hematology and Blood Transfusion, Jessenius Faculty of Medicine in Martin, Comenius University in Bratislava, 036 59 Martin, Slovakia

## Abstract

Type 2 diabetes (T2D) is associated with several abnormalities in haemostasis *predisposing* to thrombosis. Moreover, T2D was recently connected with a failure in antiplatelet response to clopidogrel, the most commonly used ADP receptor blocker in clinical practice. Clopidogrel high on-treatment platelet reactivity (HTPR) was repeatedly associated with the risk of ischemic adverse events. Patients with T2D show significantly higher residual platelet reactivity on ADP receptor blocker therapy and are more frequently represented in the group of patients with HTPR. This paper reviews the current knowledge about possible interactions between T2D and ADP receptor blocker therapy.

## 1. Introduction

Type 2 diabetes (T2D) is associated with several abnormalities in haemostasis, such as higher platelet reactivity [1, 2], endothelial dysfunction [3], and hypercoagulation and abnormalities in fibrinolysis [4], predisposing to thrombosis. ADP receptor blocker therapy is crucial in acute coronary syndrome (ACS) and postpercutaneous coronary intervention (PCI) patients to prevent future thrombotic events. According to current European Society of Cardiology and American Heart Association Clinical Practice Guidelines [5–7] ADP receptor blocker therapy should be administrated in all ST-elevation myocardial infarction (STEMI) and non-ST-elevation myocardial infarction (NSTEMI)/unstable angina (UA) patients, while in STEMI patients undergoing primary PCI new ADP receptor blockers (prasugrel, ticagrelor) should be preferred; in patients with NSTEMI/UA prasugrel should be used just when coronary anatomy is already known and a decision to perform PCI has been already established. Otherwise, ticagrelor or clopidogrel should be administrated. Moreover, these recommendations should be fully applicable in patients with as well as without T2D. Nevertheless, T2D was recently associated with a failure in antiplatelet response to clopidogrel [8, 9] which remains the most commonly used ADP receptor blocker in clinical practice [10]. Importantly, clopidogrel high on-treatment platelet reactivity (HTPR) was consistently associated with the risk of ischemic adverse events. This paper reviews the current approaches of ADP receptor blocker therapy in T2D patients.

## 2. Clopidogrel and Its Resistance in T2D Patients

Thienopyridine clopidogrel is an oral irreversible P2Y12 ADP receptor blocker. This prodrug requires oxidation by the hepatic cytochrome P450 system to generate an active metabolite. After absorption, an estimated 85% of the prodrug is hydrolysed by esterases into an inactive form, leaving only 15% of clopidogrel available for transformation to the active metabolite, which irreversibly and selectively inactivates P2Y12 ADP receptor and inhibits ADP-induced platelet aggregation [11]. The introduction of clopidogrel by the CURE study in patients with ACS [12] significantly improved the clinical outcome compared with patients treated with aspirin alone. Similar outcome was subsequently obtained in post-PCI patients [13, 14]. However, the antiplatelet effect of clopidogrel varies among individuals.

As mentioned previously, there are a growing number of data pointing to the failure in antiplatelet responses to clopidogrel which is specifically associated with insulin resistance and T2D [8, 9, 15]. These reports are based on ex vivo testing of platelet reactivity on clopidogrel therapy, as well as on subanalysis of clinical trials with clopidogrel. In these trials patients with T2D on clopidogrel therapy had worse clinical course and increased incidence of stent thrombosis [8, 9, 15–19]. The exact mechanism of this phenomenon remains currently unknown. However, the mechanism of poor clopidogrel response in T2D patients is probably multifactorial. T2D per se increases the platelet reactivity to ADP. Insulin could reduce the platelet aggregation by inhibiting the P2Y12 pathway through insulin receptors [20]. Insulin resistance might upregulate the P2Y12 ADP receptor, which is associated with clopidogrel resistance [21, 22]. An absolute or a relative lack of insulin was previously associated with increased P2Y12 signalling capacity. Moreover, this pathway appears to be in patients with T2D less sensitive to P2Y12 inhibition [23]. On the other hand, T2D may also interact with clopidogrel metabolism. T2D is already known to modulate cytochrome P450 activity in humans and in animal models [24–26]. Erlinge et al. [8] studied the prevalence and mechanism of antiplatelet failure to clopidogrel in T2D patients and in nondiabetic individuals. This double blinded study randomized totally 110 patients already treated with aspirin to clopidogrel (600 mg loading dose followed by a maintenance dose of 75 mg) or prasugrel (60 mg loading dose followed by daily maintenance dose of 10 mg) for a period of 28 days. Results of the study showed significantly higher incidence of HTPR in patients treated with clopidogrel compared to prasugrel. Diabetic patients were more frequently represented in the group with HTPR. Moreover, the HTPR was in T2D patients connected to the administration of clopidogrel. When compared with nondiabetic patients, patients with diabetes had significantly lower concentrations of clopidogrel active metabolite measured two hours after a loading dose administration (*p* < 0.01) and also on 29th day of maintenance dose usage (*p* < 0.01). It is interesting that, in this study, platelets of diabetic patients with HTPR responded well to ex vivo administration of the active clopidogrel metabolite. This observation indicates a low level of resistance on platelet P2Y12 ADP receptor and supports a potential interaction between T2D and pharmacokinetic processes of clopidogrel metabolism.

Angiolillo et al. [9] studied platelet function in diabetic and nondiabetic patients treated with aspirin and clopidogrel. Blood samples were taken after loading dose administration and on chronic therapy. The authors found significantly higher residual platelet reactivity in T2D patients both prior to clopidogrel administration and 24 hours after clopidogrel loading dose administration. In addition, the authors found a significantly higher number of patients with clopidogrel HTPR among patients with T2D. It is already known that HTPR is an independent predictor of cardiovascular events [9] and platelet reactivity on clopidogrel therapy higher than 50% was repeatedly associated with higher risk of coronary events after PCI [17, 18, 27].

The worse clinical outcome and an increased risk of ischemic events in clopidogrel-treated T2D patients were consistently demonstrated in the subanalysis of the CURE [12], CREDO [28], and Current-OASIS 7 [29] trials. These data indirectly support an incomplete response to clopidogrel associated with T2D. Additionally, Iakovou et al. [19] in an analysis of data from a prospective observational study showed that T2D is an independent predictor of stent thrombosis, despite dual antiplatelet therapy in patients after successful implantation of drug eluting stents. High frequency of clopidogrel HTPR led to the introduction of new ADP receptor blockers with more favourable pharmacodynamic profile to clinical practice.

## 3. Prasugrel: New ADP Receptor Blocker in T2D Patients

Prasugrel (Table 1) is a new thienopyridine P2Y12 ADP receptor blocker, recently introduced to clinical practice in patients with ACS and planned PCI. Prasugrel compared to clopidogrel offers more consistent inhibition of P2Y12 ADP receptor and has a lower intraindividual variability in efficacy. Prasugrel was extensively tested in the TRITON-TIMI 38 trial [30] which randomized 13 608 patients with ACS to clopidogrel or prasugrel. These patients were treated from 6 to 15 months. In this trial 3146 of patients had T2D; 776 patients were treated with insulin. The primary “endpoint” of this study was significantly decreased by prasugrel in nondiabetic group (9.2% versus 10.6%, *p* < 0.05), as well as in those with T2D (12.2% versus 17.0%, *p* < 0.001). Benefit of prasugrel administration was observed consistently in insulin-treated patients (14.3% versus 22.2%, *p* < 0.01), as well as in T2D patients without insulin therapy (11.5% versus 15.3%, *p* < 0.01). Prasugrel significantly reduced the incidence of myocardial infarction (MI) by 18% in nondiabetic subjects and by 40% in subjects with T2D. Moreover, this study showed a significant reduction of stent thrombosis by prasugrel in the overall group (0.9% versus 2.0%), as well as in T2D patients (2.0% versus 3.5%). Nevertheless, major bleeding events not associated with coronary artery bypass graft surgery occurred overall significantly more often in patients treated with prasugrel, compared to clopidogrel (2.4 versus 1.8%). In summary, throughout the study, the greatest benefit of prasugrel therapy was observed preferentially in T2D patients, in whom prasugrel significantly reduced the risk of ischemic events, including the risk of recurrent MI and the risk of stent thrombosis, without increasing the risk of serious bleeding.

On the other hand, the efficacy of prasugrel is not so convincing in patients who do not undergo invasive coronary revascularization. The TRIOLOGY ACS study [31]—a double blind, randomized prospective trial involving 7243 patients—failed to proof the significant reduction of the primary endpoint with prasugrel (10 mg daily) compared to clopidogrel (75 mg daily). Similar bleeding risk was observed in both groups of patients. In this study, 37.7% of prasugrel-treated and 38.3% of clopidogrel-treated patients had a history of T2D. Although the subanalysis of T2D patients was not reported specifically, generally there was no significant difference in the hazard ratio for primary endpoint in T2D patients compared to nondiabetic individuals (17.8% versus 11.5% in clopidogrel-treated patients, 20.4% versus 13.2% in prasugrel-treated patients, resp.; *p* = 0.71). Nevertheless, in this study, reduced ADP blocker loading doses (30 mg of prasugrel and 300 mg of clopidogrel) were administrated only in patients who underwent randomization within first 72 hours after the first medical contact and were not previously pretreated with ADP receptor blocker. Patients who did not undergo randomization within first 72 hours were treated with daily maintenance dose administration (i.e., loading dose was not administrated). This fact could influence the reduction of the primary endpoint of this study.

## 4. Prasugrel Resistance: A New Phenomenon in Diabetic Patients with ACS?

Prasugrel was repeatedly described as an effective drug for overcoming clopidogrel resistance [27, 32]. However, several recently published data reported an incomplete response to prasugrel. Prasugrel resistance might therefore become another problem in patients requiring ADP receptor blocker therapy. Silvano et al. described a rare case of resistance to both clopidogrel and prasugrel in nondiabetic patient with acute STEMI due to stent thrombosis [33]. In addition, results of recently published studies [34, 35] suggest that real prevalence of HTPR in prasugrel-treated patients may be higher than that which is traditionally considered. Bonello et al. [35] pointed out the fact that up to 25% of patients with ACS did not reach effective antiplatelet response even after 6–12 hours from prasugrel loading dose administration. There is no definite answer to the question of a possible relationship between T2D and the phenomenon of “prasugrel resistance.” We have previously described a delayed antiplatelet response to prasugrel in two T2D patients undergoing primary PCI for acute STEMI [36]. Consequently, Alexopoulos et al. [37] reported in an observational study involving 77 patients with ACS undergoing PCI that platelet reactivity in prasugrel-treated patients differed significantly by T2D status. By multivariable analysis, insulin-treated T2D was identified as the only predictor of high platelet reactivity (*p* < 0.01). The authors concluded that patients with insulin-treated T2D treated with prasugrel post-PCI have higher platelet reactivity than patients without T2D or noninsulin-treated diabetic patients. This observation supports the possible interaction between T2D and prasugrel HTPR. However, this possible interaction remains inadequately explained and further studies will be needed for the final clarification of this issue.

## 5. Cangrelor: The New Member of the ADP Receptor Blockers Family

Cangrelor (Table 1) is an intravenously administrated adenosine triphosphate analogue that binds reversibly and with high affinity to P2Y12 ADP receptor. It offers a highly effective inhibition of ADP-induced platelet aggregation immediately after administration and allows the restoration of platelet function within 1-2 hours of its discontinuation [38]. Cangrelor has been investigated in three clinical trials including a total of 24 910 patients [39–41]. A meta-analysis of these studies [42] observed a 19% risk reduction rate in periprocedural death, MI, ischemia-driven revascularization, and stent thrombosis, with a 39% risk reduction rate in stent thrombosis alone. The TIMI major and minor bleeds were increased, but there was no increase in the rate of transfusions. This new agent may be considered in ADP receptor blocker naïve patients undergoing PCI for ACS [6]. Recently, there is no study specifically investigating possible interactions between T2D and antiplatelet response to cangrelor.

## 6. Ticagrelor: A Safe and Effective ADP Receptor Blocker in T2D Patients?

Ticagrelor (Table 1) is a new oral, direct reversible P2Y12 ADP receptor blocker which achieves a higher range of inhibition of platelet aggregation compared to clopidogrel [43]. The PLATO study [44] tested the efficacy of ticagrelor and clopidogrel in the prevention of cardiovascular events in patients with ACS (totally 18.624 patients enrolled). The incidence of the primary endpoint after 12 months of follow-up was significantly lower in patients treated with ticagrelor (10.2% versus 12.3%, *p* < 0.001); there was also a significant reduction of cardiovascular deaths and stent thrombosis in the subgroup of ticagrelor-treated post-PCI patients. Ticagrelor administration was not associated with an increased risk of serious bleeding. In the group of diabetic patients ticagrelor reduced the incidence of the primary endpoint, all-cause mortality, and the risk of stent thrombosis. Similar benefit of ticagrelor therapy was seen in insulin-treated T2D patients, as well as in diabetic patients without insulin therapy. In addition, Alexopoulos et al. [45] showed significantly lower platelet reactivity in ticagrelor-treated T2D patients compared to T2D patients treated with prasugrel. Moreover, in this single-center prospective randomized study none of the T2D patients was identified as a nonresponder for ticagrelor. Consistently, ticagrelor treatment was demonstrated to be effective and even superior to prasugrel [46] in high risk diabetic patients with ACS. These data suggest that ticagrelor may be a safe and effective ADP receptor blocker in T2D patients, which can ensure consistent platelet inhibition, without the risk of HTPR, together with a good safety profile.

## 7. Detection of HTPR in Clinical Practice

**Figure 1:**
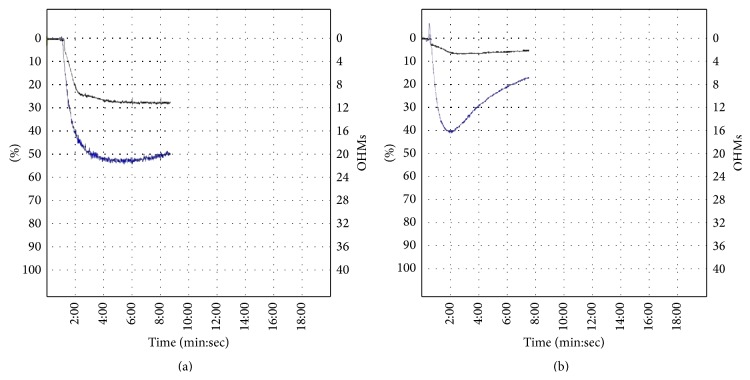
LTA with specific inducers (arachidonic acid: black curve, adenosine diphosphate: blue curve) showing difference between HTPR (a) and sufficient antiplatelet response (b) in T2D patient with acute ST-elevation myocardial infarction.

Assessing the individual level of platelet inhibition by implementing platelet function testing might help to identify patients with HTPR and therefore to reduce ischemic events. To assess the predictive level of platelet reactivity on ADP receptor blockers, numerous platelet function tests are currently available. Light transmission aggregometry (LTA) with specific inducer (adenosine diphosphate (ADP)) represents nowadays a “golden standard” in antiplatelet response testing. Maximal aggregation in response to ADP with LTA testing > 50% (Figure 1) had been associated with higher risk of ischemic events [47]. Second, vasodilator-stimulated phosphoprotein (VASP) phosphorylation flow cytometry assay represents a specific method for the assessment of ADP receptor blocker activity [48]. We have previously demonstrated that this assay is suitable for monitoring the ADP receptor blocker therapy in acute STEMI patients with primary PCI of culprit coronary lesion [49]. The advantage of this assay is its specificity for ADP receptor intracellular signaling pathway and sample stability. Nevertheless, instrumental and financial requirements may represent a possible limitation for the application of this assay in clinical practice. Third, several point-of-care assays are recently available. PFA-100 (Siemens Healthcare Diagnostics, Tarrytown, New York, USA) and Verify Now (Accumetries, San Diego, California, USA) assay methods—both based on modified aggregometry—allow quick platelet function testing in the setting of the intensive care units. Verify Now allows rapid assessment of platelet response on aspirin, P2Y12 ADP receptor antagonist, and glycoprotein IIb/IIIa antagonist treatment in one blood sample [50]. Bed site ADP receptor blockers testing may provide a rough guiding on how to proceed with treatment drugs and dosages, especially when both LTA and VASP phosphorylation assays are not available.

Although monitoring of ADP receptor blocker therapy is nowadays not generally recommended, this testing can significantly help to identify patients with HTPR. On the other hand, recently there is no definite answer to the question whether HTPR is a modifiable phenomenon. Several randomized studies trying to overcome HTPR with modified clopidogrel therapy guided by platelet function testing [51, 52] brought negative results. However, new antiplatelet agents were rarely used in these trials. Modified (increased) clopidogrel dosing, which was mostly used in these trials for overcoming the HPTR, failed to reduce the rate of major adverse cardiac events (cardiovascular death, nonfatal myocardial infarction, or stent thrombosis). The results of these randomized studies predominantly do not support a treatment strategy of high-dose clopidogrel in patients with HTPR and question the need of monitoring the on-treatment platelet reactivity in clinical practice. Nevertheless, a recently published observational study, which tested patients with planned PCI for stable angina or NSTE ACS [53], showed a reduced risk of adverse clinical events in HTPR patients with tailored intensified antiplatelet therapy. Thus, monitoring and tailoring the antiplatelet therapy might be beneficial in selected patients and deserve further investigation.

In summary, T2D seems to be associated with HTPR especially in clopidogrel-treated patients. Moreover, we have previously confirmed the association between HTPR and stent thrombosis in post-PCI patient with T2D [27]. Therefore, it is probably reasonable to routinely prefer new ADP receptor blockers over clopidogrel in T2D patients in order to ensure more effective platelet inhibition and prevent these serious thrombotic adverse events. Additionally, the subanalysis of T2D patients treated with new ADP receptor blockers did not reveal higher risk of serious bleeding. This indicates that the benefit/risk ratio is in favour of new antiplatelet agents. In case of choosing clopidogrel therapy in T2D patients, it seems to be reasonable to perform platelet function testing for the approval of sufficient on-treatment response. If this response is inadequate, the switch to new ADP receptor blocker therapy should be considered immediately. In addition, ticagrelor, in T2D patients with ACS, was demonstrated as more effective and superior even to prasugrel [46]. Thus this agent should be preferred especially in case of diabetics with acute coronary events. Nevertheless, the higher cost of medication, patient compliance, higher risk of bleeding, and other side effects should be also considered for a decision of ADP receptor blocker therapy strategy.

## 8. Conclusion

The above-mentioned evidence suggests that T2D is associated with clopidogrel HTPR. Patients with T2D show significantly higher residual platelet reactivity on clopidogrel therapy and are more frequently represented in the group of patients with clopidogrel HTPR. Moreover, several data reported that patients with insulin-treated T2D have higher residual platelet reactivity even on prasugrel therapy than patients without T2D or noninsulin-treated diabetic patients. On the other hand, ticagrelor treatment was demonstrated to be effective and even superior to prasugrel in high risk diabetic patients with ACS and ticagrelor may be a safe and effective ADP receptor blocker in these patients. However, the relationship between T2D and ADP receptor blocker therapy is not fully explained and deserves further investigation.

## Figures and Tables

**Table 1:** ADP receptor blockers in current clinical practice.

Drug	Route of administration	Bioavailability	Receptor inhibition	Time to peak platelet inhibition	Clinical application	Interactions with T2D
Clopidogrel	Oral	Prodrug	Irreversible	Highly variable	PCI, arterial interventions, ACS, stroke, and secondary prevention	Repeatedly proven
Prasugrel	Oral	Prodrug	Irreversible	2 hours	ACS with PCI	Not explicitly proven
Ticagrelor	Oral	Direct-acting	Reversible	2 hours	ACS	Probably none
Cangrelor	Intravenous	Direct-acting	Reversible	30 minutes	PCI	Not studied

ACS: acute coronary syndromes, PCI: percutaneous coronary intervention, T2D: type 2 diabetes.
